# A Novel Strategy for Accelerating Pumpable Ice Slurry Production with Ozone Micro–Nano Bubbles and Extending the Shelf Life of *Larimichthys polyactis*

**DOI:** 10.3390/foods12112206

**Published:** 2023-05-31

**Authors:** Roujia Zhang, Zhiming Cheng, Yuting Liang, Xuetao Hu, Tingting Shen, Yanxiao Li, Zhi Han, Xinai Zhang, Xiaobo Zou

**Affiliations:** 1 Instrumental Analysis Center, Jiangsu University, Zhenjiang 212013, China; 1000005191@ujs.edu.cn (R.Z.); 1000003438@ujs.edu.cn (Y.L.); 2 National Research Center of Pumps and Pumping System Engineering and Technology, Jiangsu University, Zhenjiang 212013, China; czm30913d@163.com; 3 Agricultural Product Processing and Storage Lab, School of Food and Biological Engineering, Jiangsu University, Zhenjiang 212013, China; 3190910043@stmail.ujs.edu.cn (Y.L.); xuetaojsu@ujs.edu.cn (X.H.); shentingting@ujs.edu.cn (T.S.); zou_xiaobo@ujs.edu.cn (X.Z.); 4 School of Energy and Power Engineering, Jiangsu University, Zhenjiang 212013, China

**Keywords:** pumpable ice slurry, ozone, micro–nano bubbles, nucleation, small yellow croaker, quality

## Abstract

In this study, a novel strategy for accelerating the production of pumpable ice slurry (PIS) by using ozone micro–nano bubbles (O_3_-MNBs) was proposed. The effect of PIS containing sodium alginate (SA) and O_3_-MNBs on the preservation of small yellow croaker (*Larimichthys polyactis*) was investigated. The results indicate that using SA solution containing O_3_-MNBs instead of only SA solution resulted in quicker production of PIS by promoting ice nucleation and eliminating supercooling. The distribution and positive effect of O_3_-MNBs as a nucleation agent on freezing characteristics were discussed. Microbial concentrations, pH, total volatile basic nitrogen, and thiobarbituric acid reactive substance content were also examined. Storage in novel PIS (containing O_3_-MNBs) had higher performance than storage in flake ice or conventional PIS due to the strong bacteriostatic ability of O_3_. Therefore, O_3_-MNBs injection can be used as a novel method for PIS production and the preservation of fresh marine products.

## 1. Introduction

Regarded as one of the most popular marine products in China, the small yellow croaker (*Larimichthys polyactis*) is a valuable marine resource because of its high protein and polyunsaturated fatty acid content [1,2]. After being caught, the fresh small yellow croakers, which have high water content, must be chilled with flake ice (FI). Currently, as an alternative to FI, pumpable ice slurry (PIS) is being used as the optimal cooling medium for marine products.

PIS is a novel technique for chilling and preserving the freshness of marine products in ice–water suspensions at subzero temperatures [3]. The main advantages of PIS are its (i) rapid chilling capacity, which is due to its large heat transfer surface area created by its numerous microscopic ice particles; (ii) its fluidity, which enables full coverage of the surface of the fish and mitigates dehydration and oxidation; and (iii) minor physical damage to fish, which is due to its spherical microscopic particles. By contrast, damage to fish can be caused by the sharp edges of FI [4,5].

In 2020, the aquaculture production of small yellow croakers reached 292,290 tons in China, with the scale of fishery production exhibiting an upward trend [6]. Consequently, the consumption of ice on commercial fishing vessels has considerably increased. This increased demand for cooling necessitates refrigeration systems, which consume considerable energy. Supercooling is one of the most critical problems in ice production [7]. This is because it decreases the evaporation temperature of refrigeration systems and prolongs the freezing process. The energy supply on fishing vessels is limited; thus, supercooling during the production of PIS must be controlled.

According to certain studies, micro–nano bubbles (MNBs) can be used to manipulate ice nucleation and thereby enhance the freezing process. Because of its cavitation effect, ultrasound can also be used to accelerate the freezing rate [8,9,10]. Although ice nucleation occurs spontaneously and randomly, the cavitation bubbles produced through ultrasound can promote primary heterogeneous nucleation by acting as ice nuclei [11]. In addition, pre-existing and infused MNBs can accelerate the freezing of liquid systems, such as sucrose and maltodextrin solutions [12], soft serve, milk and apple juice [13], and hydrolyzed gelatin solution [14]. Our previous research on the production of chitosan-based slurry ice demonstrated that the MNBs generated though agitation can accelerate ice nucleation and prevent supercooling [15].

PIS combined with O_3_ has been used as an effective technique for preserving marine products. Ozone is a bactericidal agent that prolongs the shelf life of fish though its oxidizing capacity and ability to reduce bacterial count [16,17]. Various marine products—such as farmed turbot [18], bighead croaker [19], tiger grouper [3], European anchovy and sardine [20], and large yellow croaker [21]—have been subjected to PIS treatment with O_3_. However, the majority of studies have focused on evaluating changes in quality during preservation; research on nucleation during the production of PIS has been insufficient.

Therefore, in the current study, a novel strategy for accelerating PIS production with O_3_-MNBs was developed, and the effect of O_3_-MNBs on the preservation of fresh small yellow croakers was also investigated. The main objectives were to (i) examine the positive effect of O_3_-MNBs on PIS production, including the O_3_-MNBs distribution and freezing characteristics, and (ii) determine the feasibility of using PIS containing O_3_-MNBs in the preservation of small yellow croakers, including calculating the preservation rate of O_3_ in PIS and freshness indicators.

## 2. Materials and Methods

### 2.1. Materials and Chemical Reagents

Sodium alginate (SA) is a hydrocolloid widely used in the food industry. SA solutions are highly viscous. In such solutions, trapped O_3_-MNBs serve as nuclei during the initiation of crystallization. Therefore, an SA solution was selected as the substrate for the preparation of PIS. In this study, 0.5 g, 1.0 g or 1.5 g SA (purchased from Sinopharm Chemical Reagent, Shanghai, China) was mixed and filled up with pure water to reach a 100 g solution. All other chemicals used were of analytical grade.

The small yellow croakers (3~4 years old, each weighing about 75 g with an average length 20~24 cm) were selected according to the sampling method [22,23]. All fish samples used in the study were from deceased fish that were purchased from local fish markets (Shenjiamen, Zhoushan, China) and then transferred to the laboratory in a portable refrigerator (0 °C). Upon arrival, the fish samples were washed with cold running distilled water.

### 2.2. Preparation of Novel PIS

**Figure 1:**
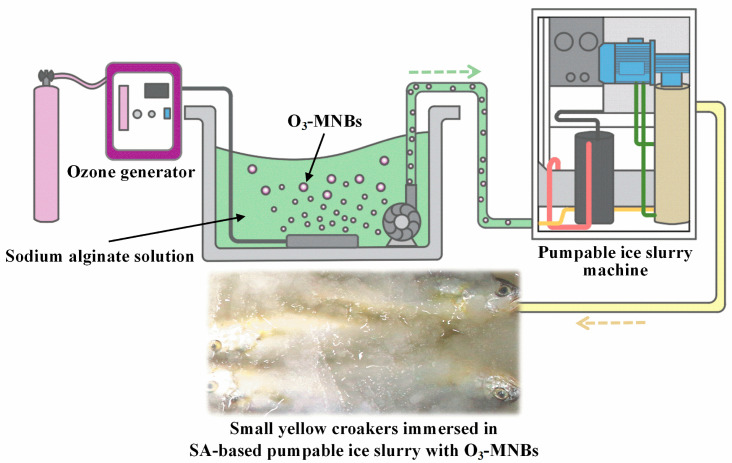
System for producing the novel PIS.

A PIS system was assembled in the laboratory (Figure 1). To improve the dissolving capacity of the hydrocolloids, the SA solution was continuously stirred in a water bath at 50 °C for 20 min and then cooled to room temperature (20 °C). An O_3_ generator (JZCF-G-3, Jiuzhoulong Company, Xuzhou, China) was subsequently employed to generate O_3_-MNBs at a concentration of 1.5 mg/L. These O_3_-MNBs were then injected into the SA solution for 15 min, and the SA solution containing O_3_-MNBs was pumped into a PIS machine (RF-1000-SP, Ruiyou Company, Nantong, China). The prepared solution was allowed to circulate though the evaporator of the PIS machine, and SA-based PIS containing O_3_-MNBs (SA-PIS-O_3_) was finally obtained after full heat exchange with the refrigerant. The temperature of the SA-PIS-O_3_ was −2 °C, and the mass ratio of the microscopic ice particles to liquid was 6:4.

### 2.3. Characterization of PIS Production

The effects of the distribution of O_3_-MNBs and the freezing behavior of SA containing O_3_-MNBs on the production of PIS were investigated. SA solutions containing 0.5, 1, and 1.5 wt% of SA power were named SA Ⅰ, SA Ⅱ, and SA Ⅲ, respectively. Consequently, the solutions containing O_3_-MNBs were named SA-O_3_ Ⅰ, SA-O_3_ Ⅱ, and SA-O_3_ Ⅲ, respectively.

#### 2.3.1. Microscopic Analysis

Before the O_3_-MNBs were pumped into the PIS machine, a light microscope (Nikon ECLIPSE Ci-L, Nikon Vision, Tokyo, Japan) was used to capture images of the O_3_-MNBs in all solution sample. The images were then analyzed using the ImageJ software (National Institutes of Health, Bethesda, MD, USA). Subsequently, the images were converted into 8-bit images, and their thresholds were adjusted. The dark areas represented the locations of the O_3_-MNBs. The light areas located at the center of the dark regions were the interiors of the bubbles, and on the images, these interiors were manually filled with dark color. The software was then used to automatically calculate the bubble count and the area of each bubble, and the bubble size distribution of each solution samples was represented as a cumulative frequency curves. For a cumulative frequency curve, steeper slop represented that the bubble size distribution was more concentrated, while long tails at the left or right side of the cumulative frequency curve indicated that the bubble size distribution was nonuniform.

#### 2.3.2. Freezing Characteristics

To determine the effects of O_3_-MNBs on the freezing behavior of SA solution during the production of PIS, the temperature of the prepared solution sample was recorded, and a freezing curve was drawn. A temperature-measuring system (Agilent 34970A, Agilent Technologies, Santa Clara, CA, USA) with a T-type thermocouple installed in the evaporator of the PIS machine was then used to measure and record the temperature of each sample every 10 s. Subsequently, time–temperature profiles were used to determine the nucleation temperature, freezing temperature, and total freezing time. The results indicate that the nucleation temperature occurred below the freezing point. Therefore, the difference between the nucleation temperature and freezing temperature was defined as the supercooling degree. In addition, the time taken for the sample to reach the freezing point from its initial temperature was defined as the total freezing time.

### 2.4. Preservation of Small Yellow Croaker

To determine whether PIS containing O_3_-MNBs can be used in the preservation of small yellow croaker, the preservation rate of O_3_ in the PIS and the freshness indicators of the fish samples were determined.

#### 2.4.1. Preservation Rate of O_3_ in PIS

The O_3_ concentrations in the solution samples (SA-O_3_ Ⅰ, SA-O_3_ Ⅱ, and SA-O_3_ Ⅲ) and PIS samples (SA-PIS-O_3_ Ⅰ, SA-PIS-O_3_ Ⅱ, and SA-PIS-O_3_ Ⅲ) were calculated as described in Yates and Stenstrom [24] using the following equation:

(1) $$O_{3}\left(\mathrm{mg}/L\right)=\frac{{\left(A_{600}\times V_{T}\right)}_{Blank}-{\left(A_{600}\times V_{T}\right)}_{sample}}{0.42\times V_{S}\times b}$$

where *A*_600_ is the sample absorbance at 600 nm, *V_T_* is the total volume (mL), *V_S_* is the sample volume (mL), and *b* is the optical path length (cm).

The preservation rate of O_3_ was calculated as follows:

(2) $$R=\frac{{\left(O_{3}\right)}_{P}}{{\left(O_{3}\right)}_{S}}\times 100\%$$

where *R* (%) is the preservation rate of O_3_, (*O*_3_)*_P_* is the O_3_ concentration in the PIS sample, and (*O*_3_)*_S_* is the O_3_ concentration in the solution sample.

#### 2.4.2. Freshness Indicators of Fish Samples

To determine the efficacy of the novel PIS in the preservation of small yellow croaker, fish samples were stored in various types of ice: (1) FI, (2) PIS, (3) SA-PIS, and (4) SA-PIS-O_3_. For Group 1, FI was prepared from distilled water with an FI machine (SM-F140AY65, Hitachi, Tokyo, Japan). For Groups 2, 3, and 4, PIS was prepared from distilled water, SA Ⅲ, and SA-O_3_ Ⅲ, respectively, by using a PIS machine. All fish samples were immersed in ice at a fish-to-ice ratio of 1:1 and then stored for up to 20 days in a refrigerated room at 0 ± 2 °C. Sampling was then performed on days 0, 4, 8, 12, 16, and 20. All experiments were conducted in triplicate.

##### Measurement of Total Plate Counts

The total viable count (TVC) of the small yellow croaker samples was determined in accordance with Chinese National Standard (GB4789.2-2016) [25]. Briefly, 5 g of treated samples was homogenized with 45 mL of 0.85% sterile NaCl solution, and a series of homogenate dilutions were then performed. After incubation at 30 °C for 72 h, the plate counting method was used to obtain the final TVC, which was expressed in log10 CFU/g.

##### Measurement of pH

The pH values of the fish samples were identified following the method reported by Wen et al. [26] with modification. A 5 g sample excised from the middle of the dorsal of the fish was thoroughly homogenized with 45 mL of distilled water. The pH of the solution was then analyzed using a pH meter (PHS-3E; INESA Scientific Instrument, Shanghai, China).

##### Measurement of Total Volatile Basic Nitrogen Content

An automatic Kjeldahl nitrogen analyzer (8400, FOSS, Denmark) was used to determine the total volatile basic nitrogen (TVB-N) content of the small yellow croaker. Briefly, a tissue sample (10 g) was mixed with 0.6 M perchloric acid solution (90 mL) and homogenized for 1 min. The mixture was then centrifuged at 10,000 rpm for 10 min (4 °C) and passed through Whatman No. 1 filter paper [27].

##### Measurement of Thiobarbituric Acid Content

The thiobarbituric acid content (TBA) of the fish samples was analyzed as described by Bensid et al. [28]. Briefly, a sample weighing 5 g was homogenized with 50 mL of 7.5% trichloroacetic acid. The homogenate was then filtered twice, and 5 mL of the supernatant was collected and mixed with TBA solution (5 mL). The solution was then heated in a water bath at 90 °C for 30 min and cooled under running tap water. Finally, a spectrophotometer (UV-1800, Shimadzu Scientific Instruments, Kyoto, Japan) was used to measure the absorbance of the cooled supernatant at 532 nm.

### 2.5. Data Analysis

All statistical analyses were performed using one-way analysis of variance in IBM SPSS Statistics version 22.0 (IBM, Armonk, NY, USA). Each experiment was conducted in triplicate. Data are expressed as the mean ± standard deviation. Statistical significance between groups was set at *p* < 0.05.

## 3. Results and Discussion

### 3.1. Bubble Distribution

**Figure 2:**
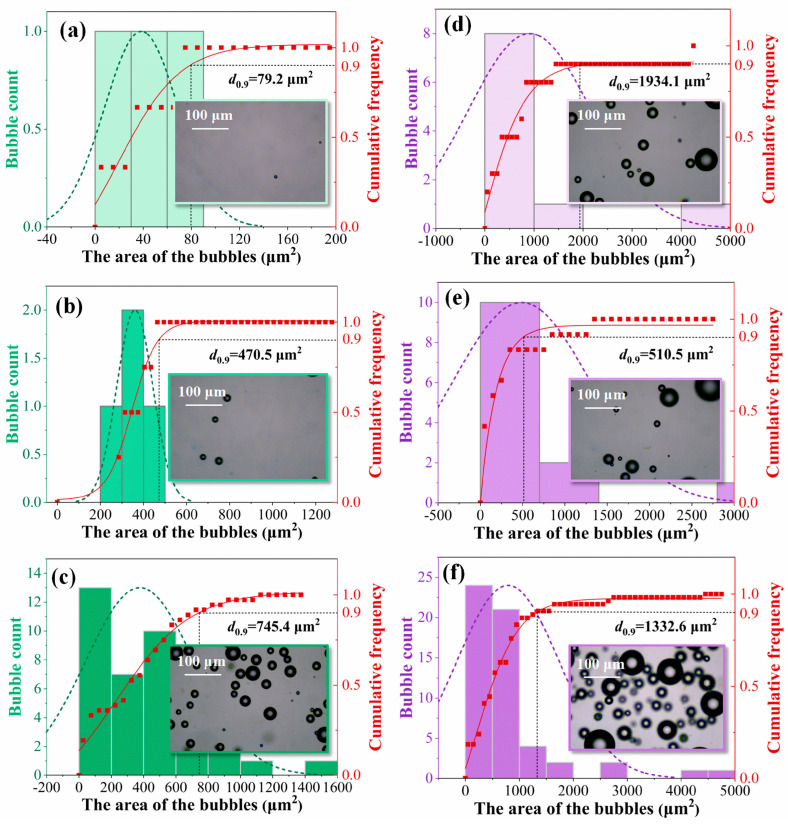
Light microscope images and bubble distribution for different samples ((**a**–**c**) are SA Ⅰ~Ⅲ, and (**d**–**f**) are SA-O_3_ Ⅰ~Ⅲ).

To determine the effect of solution concentration on the distribution of bubbles, a light microscope was used to investigate each solution sample. Figure 2a–c presents light microscope images of solutions SA Ⅰ, SA Ⅱ, and SA Ⅲ, respectively, whereas Figure 2d–f displays light microscope images of solutions SA-O_3_ Ⅰ, SA-O_3_ Ⅱ, and SA-O_3_ Ⅲ, respectively. When the solution concentration was increased, the bubble count also increased. During solution preparation, constant stirring generated bubbles in the SA samples. However, because the low-concentration SA Ⅰ sample had the weakest mutual entanglement of macromolecular chains of all the samples, it had the fewest bubbles. By contrast, compared with the other SA samples, the high-viscosity SA Ⅲ sample had the most air bubbles. In this study, the bubble count in the SA-O_3_ samples was 3.3, 3.25, and 1.48 times higher, respectively, than those in the corresponding SA samples. In addition, same as for the SA samples, the SA-O_3_ samples had higher bubble density when the solution concentration was higher, with the bubble count increasing from 10 to 13 and 55. This bubble count result is consistent with our previous work [15] showing that solutions with higher viscosity could incorporate more air microbubbles, which are generated though agitation. Because the mutual entanglement of the macromolecular chains was strong, the SA samples with higher concentrations had higher viscosity, leading to more bubbles becoming trapped [29].

Representing the distribution of bubble size as a cumulative frequency curve is an intuitive method for visualizing the results. As shown in Figure 2, the size of 90% of the bubbles ranged from 79.2 to 745.4 μm^2^ in the SA samples and from 510.5 to 1934.1 μm^2^ in the SA-O_3_ samples. Comparable observations were also made in our previous work [15] that a number of approximately micron-sized air bubbles existed and were effective in accelerating slurry ice production. However, the size of the infused bubbles varies considerably from document to document. Adhikari et al. [13] reported that the CO_2_ nanobubbles of around 100 nm diameter in range were effective in promoting the freezing properties of soft serve, milk, and apple juice. Additionally, Xu et al. [30] reported that a great many micron-sized bubbles can be obviously observed in gelatin gel samples, which can shorten the nucleation delay, but some bubbles were above centimeter level. In this study, although the bubbles in the SA samples were larger when the solution concentration was larger, this trend was not observed in the SA-O_3_ samples. As illustrated in Figure 2d,f, the SA-O_3_ Ⅰ sample had larger bubbles than those in the SA-O_3_ Ⅲ sample. In the SA samples, the air bubbles were generated though agitation and trapped in the solutions. For the SA-O_3_ samples, O_3_ was injected into the SA solutions; with this injection, the pre-existing air bubbles and the O_3_-MNBs would coalescence or breakup during the bubble rising period and result in changes in bubble size distribution [31]. This outcome is consistent with the finding of Tian et al. [14], who reported that severe coalescence of nanobubbles might occur in lower-concentration hydrolyzed gelatin due to low viscosity. However, the number of bubbles in the SA-O_3_ Ⅲ sample was considerably higher than that in the SA-O_3_ Ⅰ sample. In this study, O_3_ is infusible and is highly likely to be present in the form of O_3_-MNBs in SA samples. These O_3_-MNBs can serve as ice nuclei in the subsequent production of PIS. Additionally, the effect of O_3_-MNBs on SA solution during PIS production is discussed in the following sections.

### 3.2. Freezing Characteristics of SA and SA-O_3_ Solutions

**Figure 3:**
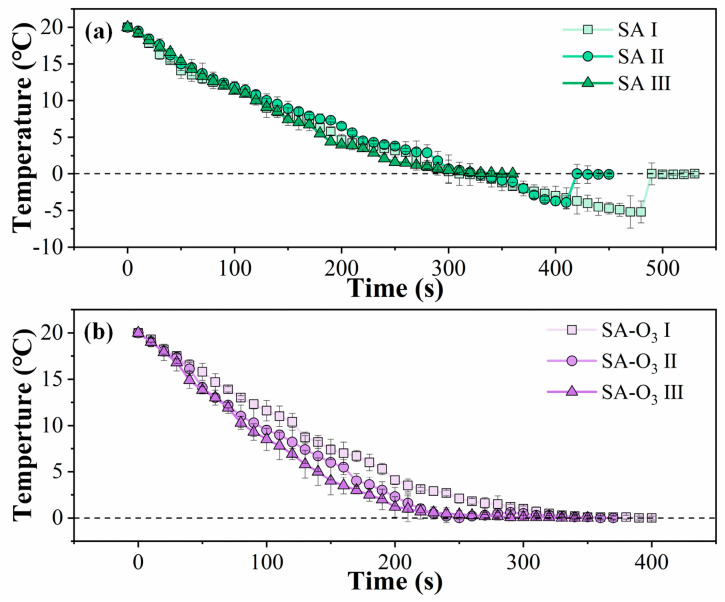
Time–temperature profiles for (**a**) SA samples and (**b**) SA-O_3_ samples during PIS production.

Figure 3 depicts the freezing curves obtained when the SA and SA-O_3_ samples were used to produce PIS. Supercooling behavior was clearly observed for the SA Ⅰ and SA Ⅱ samples. The temperature fell below the freezing point without nucleation, and the freezing curves then sharply rose after the initiation of nucleation. After the latent heat in the samples had been released, ice crystals gradually grew in the solutions. Subsequently, PIS containing multitudes of ice crystals was continually generated along with liquid solution in the PIS machine. The SA Ⅰ sample resulted in the longest freezing process, along with a high supercooling degree. In addition, the nucleation temperature for the SA Ⅱ sample was higher than that for the SA Ⅰ sample. Of the SA samples, the SA Ⅲ sample nucleated at the highest temperature (at approximately 0 °C), which was due to the presence of numerous air bubbles. As shown in Figure 3b, a similar freezing trend to that for SA Ⅲ was discovered for all SA-O_3_ samples. However, no supercooling behavior was observed, and the freezing period gradually decreased as the solution concentration was increased. These results are expected because the O_3_-MNBs that formed as a result of O_3_ infusion served as ice nuclei, thereby overcoming the nucleation barrier. Generally, in a liquid system, each bubble is regarded as a potential ice nucleation site and leads to the formation of additional smaller crystals [31]. In this study, the SA-O_3_ Ⅲ sample had a sufficient number of ice nucleation sites, enabling the rapid formation of PIS.

Table 1 lists the nucleation temperature, supercooling degree, and total freezing time of the PIS produced using the SA samples with and without O_3_-MNBs. The nucleation temperature of the SA Ⅰ sample was −5.2 ± 1.4 °C; the nucleation temperatures of the SA Ⅱ sample, SA Ⅲ sample, and all SA-O_3_ samples were significantly higher than this value (*p* < 0.05). Considerable deviation was discovered in the nucleation that occurred, indicating the spontaneous and random nature of the nucleation. The supercooling degrees of the SA Ⅱ sample was 25% lower than those of the SA Ⅰ sample. Additionally, for the SA Ⅲ samples and all the SA-O_3_ solutions, supercooling was eliminated. In addition, the total freezing time was considerably shorter for the SA-O_3_ samples than for the SA samples with corresponding concentrations. The freezing rate increased with the solution concentration of the SA and SA-O_3_ samples, which is in accordance with the bubble size distribution results. Specifically, the total freezing times of the SA Ⅱ and SA Ⅲ were 14.07% and 27.47%, respectively, shorter than that of the SA Ⅰ sample, and the total freezing times of the SA-O_3_ Ⅱ and SA-O_3_ Ⅲ were 8.42% and 20.17%, respectively, shorter than that of the SA-O_3_ Ⅰ sample. Because the SA-O_3_ samples contained more bubbles than the SA samples, these samples nucleated at 0 °C, with a shorter freezing period. These results confirm that supercooling occurred during the freezing of the SA solutions in the PIS machine. They also indicate that the introduction of O_3_-MNBs considerably increased the freezing rate. Thus, the introduction of nucleation seeds reduced the nucleation barrier of the solution system, and any small reduction in the nucleation barrier can result in considerable differences in the nucleation rate and the total freezing time [32]. Although the SA-O_3_ Ⅰ and SA-O_3_ Ⅱ samples had similar bubble count, the freezing time of SA-O_3_ Ⅱ was shorter than that of SA-O_3_ Ⅰ. This conflict might be due to the fact that the O_3_-MNBs in SA-O_3_ Ⅰ were larger than those in SA-O_3_ Ⅱ, which suggested that the contact angle of the nucleus in the SA-O_3_ Ⅰ sample was closer to 180°, which resulted in a higher activation barrier [33]. The results are similar to those obtained for air bubbles in liquid samples [15,34], bubbles infused in gel food systems [30], and cavitation bubbles generated during ultrasound-assisted freezing processes [14,35].

Overall, the high degree of supercooling can serve as a driving force for nucleation, and the region around the bubbles is where crystallization occurs first [36,37]. This means that O_3_-MNBs can be added to liquids to accelerate the production of PIS and trigger nucleation. In the following sections, the effect of O_3_-MNBs on the preservation of fish is discussed.

### 3.3. Preservation of Small Yellow Croaker

#### 3.3.1. Rate of O_3_ Preservation

**Figure 4:**
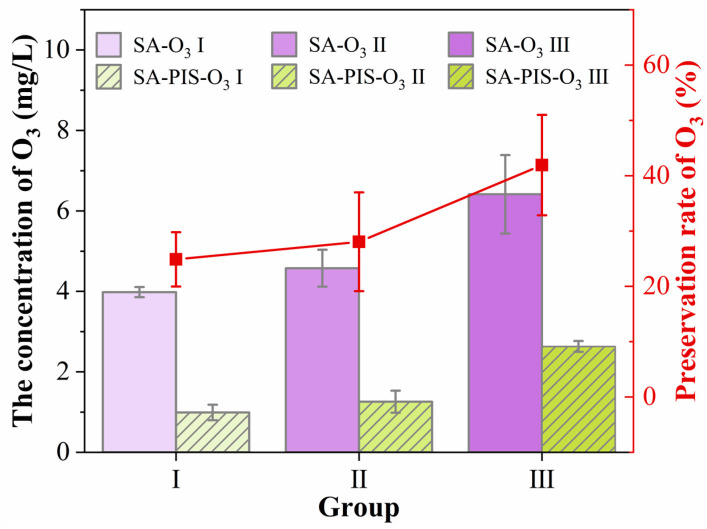
The concentration of O_3_ in SA-O_3_ samples and SA-PIS-O_3_ samples.

Figure 4 depicts the results for the O_3_ preservation rate in the PIS samples. When the SA concentration was increased, the O_3_ concentration in the solution samples (SA-O_3_ Ⅰ, SA-O_3_ Ⅱ, and SA-O_3_ Ⅲ) and SA-PIS-O_3_ samples (SA-PIS-O_3_ Ⅰ, SA-PIS-O_3_ Ⅱ, and SA-PIS-O_3_ Ⅲ) gradually increased, consistent with the bubble distribution results. Specifically, the concentration of O_3_ in the SA-O_3_ Ⅰ, SA-O_3_ Ⅱ, and SA-O_3_ Ⅲ were determined to be 3.98 ± 0.12 mg/L, 4.58 ± 0.46 mg/L, and 6.41 ± 0.97 mg/L, respectively, whereas in the SA-PIS-O_3_ samples, lower values of 0.99 ± 0.19 mg/L, 1.26 ± 0.27 mg/L, and 2.63 ± 0.13 mg/L, respectively, were obtained. This result was observed because the specific surface area of the PIS was large; the O_3_-MNBs trapped in SA-O_3_ samples were released easily during the production of PIS. This phenomenon has also been clarified by Matsumoto et al. [38], where the concentrations of ozone microbubbles in the ice with larger dimensions were kept higher. Compared with whole ice (80 × 80 mm^2^) and cut ice (20 × 20 mm^2^), ozone microbubble concentration for the crushed ice (4 × 4 mm^2^) was minimal. In this study, when the SA solution concentration was increased, the O_3_ preservation rate also increased. This is because trapping O_3_-MNBs in PIS was much easier when the substrate solution was more viscous due to more entanglements between the polymer chains [29].

Although all batches had a low O_3_ preservation rate (ranging from 24.9% to 41.9%) in this study, the results obtained in this study are sufficient within the context of pasteurization [39]. Matsumoto et al. [38] confirmed that a pseudoice slurry (formed by mixing crushed ice containing microbubbles with pure water) was effective for the cold storage, sterilization, and deodorization of food. PIS can be used to effectively and rapidly chill fish products thanks to its high fluidity and large specific surface area. In the following sections, specific freshness indicators are discussed for small yellow croakers stored in the novel PIS which contained O_3_-MNBs.

#### 3.3.2. Freshness Indicators

##### Microbiological Analysis

**Figure 5:**
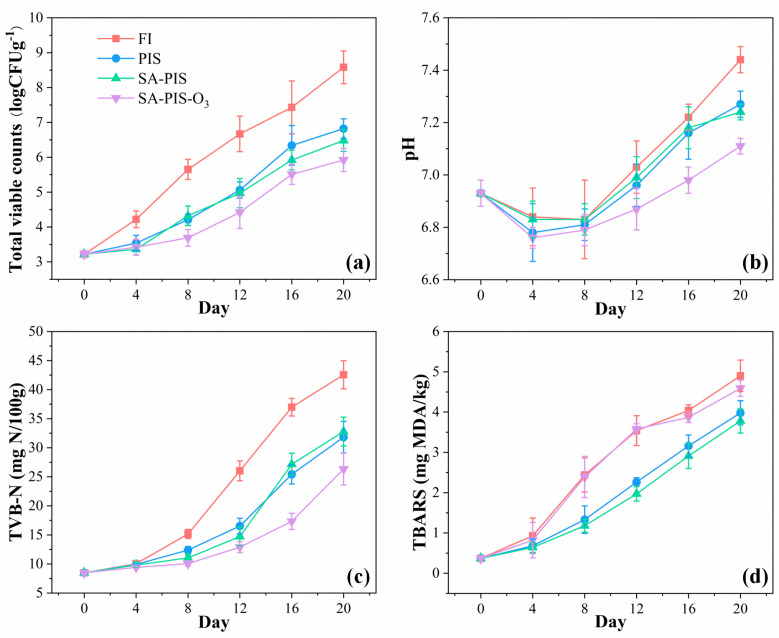
(**a**) TVC, (**b**) pH, (**c**) TVB-N, and (**d**) TBARS of small yellow croaker during storage.

Approximately 30% of fish caught lose their value as an edible product as a result of microorganism activity [40]. In this study, changes in the TVC of small yellow croaker were monitored during its storage in FI, PIS, SA-PIS, and SA-PIS-O_3_, as shown in Figure 5a. The initial TVC of the fresh small yellow croaker was 3.22 ± 0.13 log CFU/g. Preservation in FI resulted in substantially larger microbial populations and a higher TVC (6.67 ± 0.51 log CFU/g) after 12 days of storage than did storage in the other types of ice. For these other three batches, microbial growth was considerably lower, with the TVC reaching only up to 5.06 ± 0.23 log CFU/g, 4.97 ± 0.42 log CFU/g, and 4.42 ± 0.46 log CFU/g, respectively, after 12 days. According to the International Commission on Microbiological Specifications for Foods (ICMSF) [41], the maximum TVC permitted for freshwater and marine products is 7.0 log CFU/g, which indicates that the products are suitable for consumption. However, in this study, the FI batch had a TVC larger than this threshold; it reached 8.58 ± 0.47 log CFU/g at the end of the storage period. Compared with FI, which could only cool the aquatic products to around 0 °C, PIS is a ideal cooling medium which could cool the aquatic products at −0.5 to −1.5 °C [19]. Moreover, PIS can cover the surface of the fish samples fully due to its good fluidity. Thus, PIS has been verified to be effective in suppressing bacterial growth and other chemical reactions. Similarly, in this study, after 20 days of storage, the TVC values obtained for the PIS, SA-PIS, and SA-PIS-O_3_ batches were below the suggested threshold. However, the TVCs of the samples placed in PIS did not significantly differ (*p* > 0.05) from those of the samples that were placed in SA-PIS, while for the SA-PIS-O_3_ batch, the TVC of small yellow croaker only underwent a slight increase; the TVC at the end of storage was 5.92 ± 0.33 log CFU/g. Similar results were reported by Zhao et al. [21], who placed large yellow croaker (Pseudosciaena crocea) in ozonated ice slurry (which comprised a mixture of O_3_ water and NaCl). Compared with the FI and slurry ice batches, the large yellow croakers placed in the ozonated ice slurry had a clearly lower TVC. This was because O_3_ is a powerful sanitizer with a strong oxidation effect [42]. In this study, the SA-PIS-O_3_ used was a triphase mixture of ice microcrystals, SA solution, and O_3_, and the O_3_-MNBs effectively remained or dissolved in the high-viscosity SA solution. This means that the novel PIS considerably inhibited the growth of microorganisms.

##### pH

Figure 5b depicts the changes in the pH values of the four batches placed in FI, PIS, SA-PIS, and SA-PIS-O_3_. When the fish samples arrived at our laboratory (day 0), no considerable pH differences were discovered between the four batches, and the initial pH was 6.93 ± 0.05. However, after 4 days of storage, the pH had decreased to 6.84 ± 0.11, 6.78 ± 0.11, 6.83 ± 0.07, and 6.76 ± 0.04. The pH then slightly increased, but no considerable differences were found between the four batches until day 12. In all cases, the pH of the fish samples remained below 7. The initial decrease is due to the production of lactic acid from the hydrolysis of the glycogen after death [39]. Thereafter, the pH gradually increased to 7 and above, and the FI batches had considerably higher pH values than did the other batches. At the end of the storage period for the fish samples stored in FI, the pH reached 7.44 ± 0.05, whereas it ranged from 7.11 to 7.27 in the other batches. This was most likely due to the undesirable nitrogen-containing compounds produced by the bacterial action [43]. Of all the batches, the SA-PIS-O_3_ batches had the lowest pH, indicating that this novel strategy effectively slowed the protein degradation process and delayed the deterioration of fish samples’ quality. Similar results were obtained in Campos et al. [44], which reported that the pH values of the sardine (*Sardina pilchardus*) stored in ozonated slurry ice was significantly lower than that of sardine stored in FI or slurry ice. Therefore, according to the present results, the introduction in O_3_-MNBs may inhibit the alkalization of microflora during the preservation of fish.

##### TVB-N

TVB-N content is regarded as a useful indicator of the quality of marine fish products during their storage [45]. Figure 5c depicts the changes in the TVB-N content. In the FI batch, the TVB-N content rapidly increased from an initial value of 8.48 ± 0.29 mg/100 g and exceeded the upper limit (30 mg/100 g, concentrations above which are considered unsuitable for consumption) after 12 days of storage. However, the rate of increase in TVB-N content in the PIS, SA-PIS, and SA-PIS-O_3_ batches was slower than that of FI batch. These results are in line with the results reported by Yuan et al. [27], indicating that a large number of amino acids (e.g., methionine and tyrosine) were destroyed when the fish samples were stored in FI compared with when they were stored in slurry ice. According to the SC/T 3101-2010 standard, fresh small yellow croaker with a TVB-N content of less than 13 mg/100 g is considered to be of superior grade, whereas 13~30 mg/100 g is regarded as edible but slightly decomposed. In this study, only the SA-PIS-O_3_ batch remained at a fresh level after being stored for 12 days. At the end of the storage period, the TVB-N content of the FI batch was 42.55 ± 2.42 mg/100 g, whereas the PIS, SA-PIS, and SA-PIS-O_3_ batches exhibited considerably lower TVB-N contents of 31.8 ± 2.71 mg/100 g, 32.78 ± 2.49 mg/100 g, and 26.32 ± 2.73 mg/100 g, respectively. However, no significant differences in TVB-N content were discovered between the PIS and SA-PIS batches. Notably, although the TVB-N content of the PIS and SA-PIS batches slightly exceeded 30 mg/100 g; the TVB-N content of the SA-PIS-O_3_ batches remained below the threshold. These results indicate that the injection of O_3_-MNBs may effectively impede the formation of TVB-N. Overall, these results agree with those obtained for bighead croaker [19] and sardine [44]. In these cases, the TVB-N content in the FI group rapidly increased during the storage period, and the fish samples stored in ozonated slurry ice had the lowest lipid hydrolysis rate.

##### TBA Reactive Substances

Figure 5d depicts the changes in the TBA reactive substances (TBARS) content of the small yellow croaker during its storage. The initial TBARS content of the fresh fish samples was 0.37 ± 0.02 mg MDA/kg, and this content increased during the storage period for all batches. Compared with the PIS and SA-PIS batches, the FI batch had significantly higher TBARS content from day 4, which was in line with the trends in the other freshness indicators investigated in this study (TVC, pH, and TVB-N). Specifically, in the FI batch, the TBARS content had considerably increased to 4.9 ± 0.39 mg MDA/kg by the end of the storage period, whereas in the PIS and SA-PIS batches, the TBARS content was 3.98 ± 0.3, and 3.78 ± 0.3 mg MDA/kg, respectively. According to Binsi et al. [46], a concentration of 2 mg/100 g is typically regarded as the upper permissible limit of TBARS content in fresh fish muscle. In this study, the TBARS content in the FI batch exceeded the limit on day 8, whereas the PIS and SA-PIS batches remained fresh states with TBARS content of 1.33 ± 0.34 and 1.18 ± 0.16 mg MDA/kg, respectively. These results indicate that PIS can slow the secondary oxidation process. However, the trend observed in the SA-PIS-O_3_ batch was similar to that in the FI batch, which exhibited higher TBARS content than did the PIS and SA-PIS batches from day 4. Similar trends were also observed by Zhao et al. [21], who reported that the TBARS content of fish samples stored in ozone slurry ice was relatively high. The lipid oxidation in the SA-PIS-O_3_ batch might be accelerated by the oxidizing action of ozone. The injection of O_3_-MNBs was effective in terms of pasteurization but would also oxidize and destroy the fat of the fish samples.

Table 2 lists a comparison between previously reported works and this work. Storage of the aquatic products in PIS combined with O_3_ improves microbiological and biochemical quality as compared with storage in FI. However, the TVB-N trends in our work are not consistent with the results of Zhao et al. [21] and Liu et al. [47], who reported that the TVB-N content had a downward trend during the storage period. The decrease in TVB-N in these results may be due to the volatilization of TVB-N in fish into the melting ice water. In addition, the combination of O_3_ led to a negative effect that accelerated fat oxidation in the fish samples. However, it was found that the TBARS values of all batches in our work were less than 8.0 mg MDA/kg at the end of the storage, which was considered the acceptable limit for aquatic products [21].

## 4. Conclusions

In this study, the effect of O_3_-MNBs on the production of PIS was investigated. Additionally, a series of microbial and biochemical parameters were also employed to determine whether O_3_-MNBs combined with PIS are effective for the preservation of small yellow croaker. The results indicate that the injection of O_3_-MNBs eliminated the supercooling effect observed during the production of PIS and shortened its total freezing time over 7.7%. Because each bubble is generally regarded as a potential nucleation site during the production of PIS, the SA solutions with more O_3_-MNBs had a higher nucleation rate, thereby resulting in a shorter freezing time. The relationship between bubble distribution and freezing characteristics was also discussed. In addition, our results indicate that the injection of O_3_-MNBs has positive effects on fish preservation, such as maintaining bacterial count and pH and TVB-N. However, it is worth noting that the injection of O_3_-MNBs would increase the fat oxidation of the fish samples (TBARS content up to 4.59 mg MDA/kg at the end of storage). Therefore, more types of PIS combined with antimicrobial compounds should be developed in the future.

## Figures and Tables

**Table 1:** Nucleation temperature, supercooling degree, and total freezing time for different samples during PIS production.

Solution	Nucleation Temperature (°C)	Supercooling Degree (°C)	Total Freezing Time (s)
SA Ⅰ	−5.2 ± 1.4 c	5.2 ± 1.4	473.3 ± 20.8 a
SA Ⅱ	−3.9 ± 0.9 b	3.9 ± 0.9	406.7 ± 5.8 b
SA Ⅲ	0.0 ± 0.3 a	/	343.3 ± 5.8 cd
SA-O_3_ Ⅰ	0.0 ± 0.2 a	/	396.7 ± 11.5 b
SA-O_3_ Ⅱ	0.0 ± 0.2 a	/	363.3 ± 5.8 c
SA-O_3_ Ⅲ	0.0 ± 0.2 a	/	316.7 ± 5.8 d

Different lowercase letters (a–d) in the same column indicate significant differences in different samples (*p* < 0.05).

**Table 2:** A comparison between previously reported works and this work.

Ice Type	Fish Sample	Storage Time	Freshness Indicators				Reference
			TVC (log CFU/g)	pH	TVB-N (mg/100 g)	TBARS (mg MDA/kg)	
FI	squid	Day 15	6.61	-	40	-	[27]
	mackerel	Day 21	>7.0	7.2	31.18	3.8	[47]
	large yellow croaker	Day 21	8.7	7.42	25.82	3.5	[21]
PIS	farmed perch	Day 20	5.60	-	32.5	-	[48]
	squid	Day 15	4.74	-	13.26	-	[27]
PIS combined with slightly acidic electrolyzed water	mackerel	Day 24	5.8	6.58	7.5	2.51	[47]
PIS combined with chitosan	silver pomfret	Day 20	5.28	6.76	24.78	4.28	[15]
PIS combined with O_3_	sardine	Day 22	-	6.07	32.22	-	[18]
	large yellow croaker	Day 21	6.85	7.28	7.09	3.10	[21]
	small yellow croaker	Day 20	5.92	7.11	26.32	4.59	This work

## Data Availability

Data is contained within the article.
